# Effects of imeglimin on liver enzymes and body composition in patients with MASLD and type 2 diabetes: a pilot study

**DOI:** 10.1007/s13340-026-00931-4

**Published:** 2026-10-05

**Authors:** Kaede Fujita, Takanobu Iwadare, Takefumi Kimura, Yuma Kamijo, Taiki Okumura, Shun-ichi Wakabayashi, Hiroyuki Kobayashi, Tomoo Yamazaki, Naoki Tanaka, Hideo Kunimoto

**Affiliations:** 1 Department of Hepatology, Nagano Municipal Hospital, Nagano, Japan; 2 Department of Medicine, Division of Gastroenterology, Shinshu University School of Medicine, Matsumoto, Japan; 3 Consultation Center for Liver Diseases, Shinshu University Hospital, Matsumoto, Japan; 4 Institute for Biomedical Sciences, Research Cluster for Social Implementation, Matsumoto, Japan; 5 Department of Global Medical Research Promotion, Shinshu University Graduate School of Medicine, Matsumoto, Japan; 6 International Relations Office, Shinshu University School of Medicine, Matsumoto, Japan; 7 Research Center for Social Systems, Shinshu University, Matsumoto, Japan

**Keywords:** Imeglimin, Metabolic dysfunction-associated steatotic liver disease, Type 2 diabetes

## Abstract

**Background:**

Metabolic dysfunction-associated steatotic liver disease (MASLD) is highly prevalent in patients with type 2 diabetes mellitus (T2DM), yet effective pharmacological options remain limited. Imeglimin, a novel antidiabetic agent targeting mitochondrial bioenergetics, has shown potential hepatoprotective effects in preclinical studies; however, its impact on liver function and body composition in MASLD remains unclear.

**Methods:**

This retrospective, single-center pilot study analyzed prospectively collected data from 28 patients with MASLD and T2DM who were treated with imeglimin for 6 months. Clinical and biochemical parameters were assessed at baseline and at 3 and 6 months. Body composition was evaluated using bioelectrical impedance analysis.

**Results:**

Over 6 months, fasting blood glucose decreased from 148.5 to 131.0 mg/dL (*p* = 0.015), accompanied by reductions in liver enzymes (aspartate aminotransferase: 33–26 U/L, *p* < 0.001; alanine aminotransferase: 28.5–23.5 U/L, *p* = 0.001). Body weight and body fat percentage also decreased (*p* < 0.001 and *p* = 0.018, respectively), whereas no statistically significant changes were observed in skeletal muscle mass. No serious adverse events were reported.

**Conclusion:**

Imeglimin therapy was associated with favorable changes in glycemic parameters, liver enzymes, and body composition in this pilot cohort, without a statistically significant reduction in skeletal muscle mass. These findings suggest a potential metabolic and hepatic effect of imeglimin and may be particularly relevant for patients with MASLD at risk of sarcopenia. Larger prospective studies with histological or validated non-invasive endpoints are warranted.

**Graphical abstract:**

**Figure:**
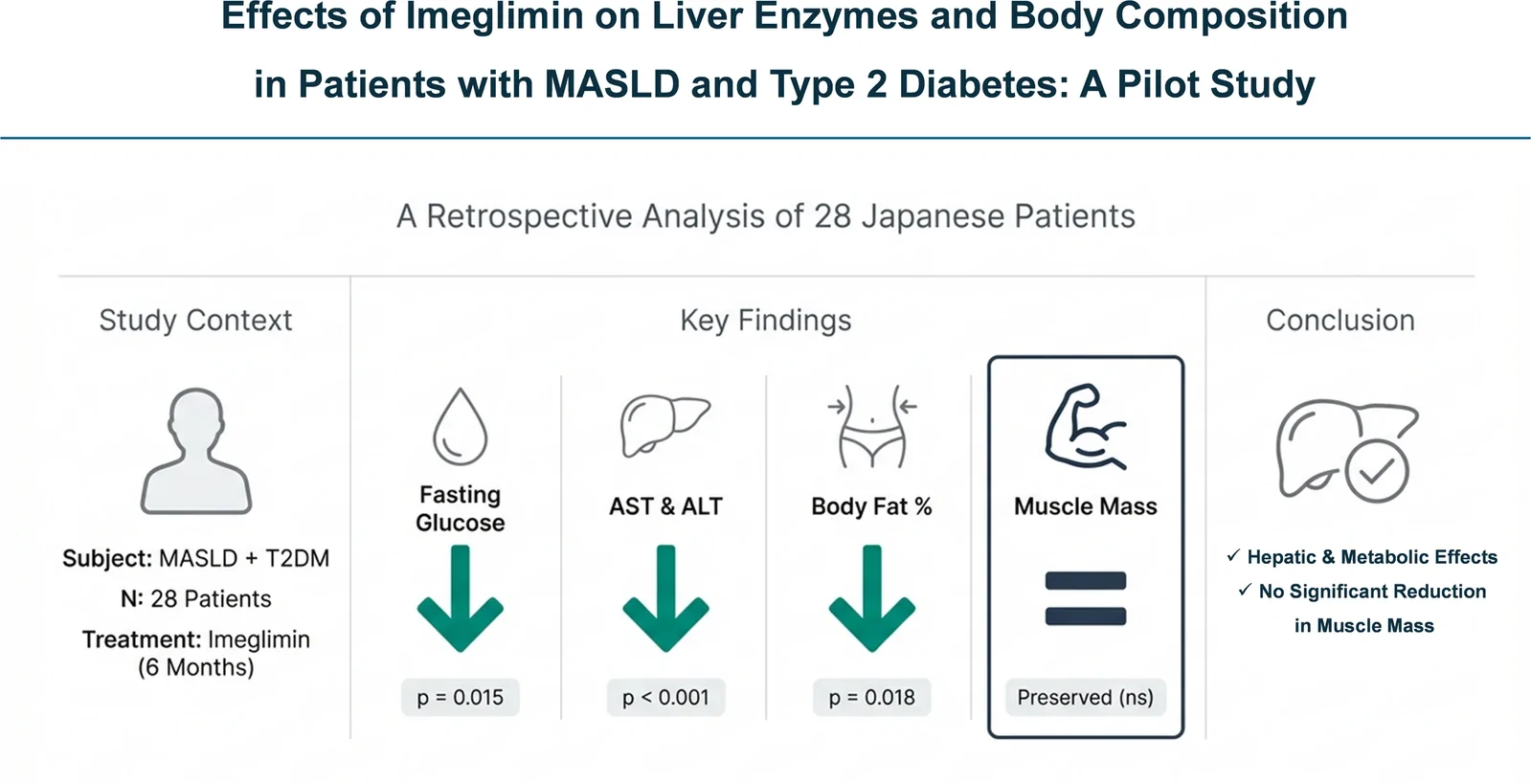

**Supplementary Information:**

The online version contains supplementary material available at https://doi.org/10.1007/s13340-026-00931-4.

## Introduction

Steatotic liver disease comprises a spectrum of hepatic disorders characterized by excessive lipid accumulation in hepatocytes, encompassing conditions formerly classified as non-alcoholic fatty liver disease (NAFLD) and alcoholic liver disease [1]. In recent years, NAFLD has been redefined as metabolic dysfunction-associated steatotic liver disease (MASLD), which is diagnosed in the presence of hepatic steatosis together with at least one metabolic abnormality, such as obesity, type 2 diabetes mellitus (T2DM), hypertension (HT), or hyperlipidemia (HL) [1, 2]. Among these metabolic factors, T2DM is particularly strongly associated with liver fibrosis progression and adverse clinical outcomes in patients with MASLD [3, 4].

MASLD is now one of the most prevalent chronic liver diseases worldwide and is associated not only with cirrhosis and hepatocellular carcinoma [5, 6], but also extrahepatic complications including cardiovascular disease and such malignancies as colorectal cancer [7–9]. Although lifestyle modification remains the cornerstone of MASLD management, the pharmacological landscape for MASH has recently evolved. Resmetirom has been approved for noncirrhotic MASH with moderate-to-advanced fibrosis in several countries [10], while semaglutide has recently been approved in Japan for noncirrhotic MASH with moderate-to-advanced fibrosis [11]. Beyond these disease-specific therapies, other GLP-1 receptor agonists (GLP-1RAs) and sodium-glucose cotransporter-2 (SGLT2) inhibitors are widely used for the treatment of obesity and/or type 2 diabetes mellitus and have demonstrated beneficial effects on hepatic steatosis and metabolic parameters [12, 13]. However, reductions in lean mass, including skeletal muscle mass, have been reported with these therapies, raising concerns about potential muscle loss in patients at risk of sarcopenia [14–16].

Imeglimin is a novel oral anti-diabetic agent approved for clinical use in Japan in 2021. It improves glucose homeostasis through complementary mechanisms, including improvement of mitochondrial function, enhancement of glucose-stimulated insulin secretion, suppression of excessive hepatic gluconeogenesis, and improvement of insulin sensitivity [17, 18]. Although large-scale clinical trials are lacking, early evidence from animal models suggests that this agent may also modulate hepatic lipid metabolism through mitochondrial pathways [19]. Given its mitochondrial mechanism and favorable metabolic profile, imeglimin may favorably influence body composition by reducing fat mass without compromising muscle mass, an effect of clinical relevance in patients with MASLD at risk of sarcopenia [20]. This study aimed to evaluate the effects of imeglimin on liver function, glycemic control, and body composition in patients with MASLD and T2DM.

## Materials and methods

### Patients and clinical examinations

This study was a retrospective analysis of prospectively collected data and was approved by Nagano Municipal Hospital (approval no. 0059). All procedures were conducted in accordance with the Declaration of Helsinki (1975, revised in 1983). We reviewed the medical records of 28 Japanese patients with MASLD and concomitant T2DM who were treated with imeglimin at Nagano Municipal Hospital (Nagano, Japan) between November 2021 and March 2025. Therapeutic effects were assessed using laboratory data obtained at baseline and at 3 and 6 months after initiation of imeglimin. Imeglimin was administered orally at a dose of 1000 mg twice daily (morning and evening) throughout the 6-month study period. Throughout the observation period, all patients received standard-of-care dietary and exercise counseling. Registered dietitians and physicians provided dietary and physical activity guidance in accordance with the recommendations of the Japan Diabetes Society for the management of type 2 diabetes [21]. Lifestyle counseling was delivered as part of routine clinical care; its frequency and intensity were not prespecified or standardized. Medication adherence was assessed by patient self-report at each outpatient visit, whereas adherence to lifestyle recommendations was not systematically monitored.

The inclusion criteria were: (1) ultrasonographic evidence of hepatic steatosis, defined by increased hepatic echogenicity and hepatorenal contrast; and (2) metabolic dysfunction fulfilling the international diagnostic criteria for MASLD [22], at a minimum represented by the presence of T2DM, defined according to standard criteria (fasting plasma glucose ≥126 mg/dL, HbA1c ≥6.5%, 2-h plasma glucose ≥200 mg/dL during an oral glucose tolerance test, or current treatment with insulin or oral hypoglycemic agents) [21]. The final analysis cohort consisted of patients with MASLD and T2DM who completed the scheduled follow-up. The main exclusion criteria were other causes of liver disease, including viral hepatitis, drug-induced liver injury, autoimmune liver disease, Wilson’s disease, hereditary hemochromatosis, and citrin deficiency [23]. None of the participants met the alcohol consumption thresholds for metabolic dysfunction–associated alcohol-related liver disease or alcohol-associated liver disease, and none had a history of exposure to suspected hepatotoxic agents. Hypertension was defined as systolic/diastolic blood pressure ≥140/90 mmHg or current use of antihypertensive medication [24]. Hyperlipidemia was defined as fasting total cholesterol ≥ 220 mg/dL, low-density lipoprotein cholesterol ≥ 140 mg/dL, triglycerides ≥ 150 mg/dL, or current use of lipid-lowering therapy [25].

Serum alanine aminotransferase (ALT) was pre-specified as the primary endpoint. Secondary endpoints included other liver-related biochemical parameters, glycemic indices, and changes in body composition. Patients with a  ≥ 30% reduction in ALT from baseline at 6 months were classified as responders, according to criteria used in previous interventional studies of MASLD/NAFLD [26–28]. All laboratory measurements were performed in the fasting state. The albumin–bilirubin (ALBI) score was calculated using the established formula [29]. Blood samples were obtained every 4–12 weeks, and adverse events were systematically assessed at each visit by structured interviews and routine laboratory evaluation.

### Body composition analysis

Body composition analysis was conducted using an InBodyS10 multi-frequency impedance body composition analyzer (InBody Japan, Tokyo). The analysis included measurements of fat mass, soft lean mass, and skeletal muscle mass. Body composition was assessed under fasting conditions during routine outpatient visits. Because of the retrospective study design, measurement timing and resting period before assessment were not standardized. Measurements were performed as part of routine clinical care when measurement slots were available. Accordingly, paired body composition measurements at baseline and 6 months were available for 15 patients.

### Statistical analysis

Clinical data are expressed as number (percentage) or median (interquartile range [IQR]). Statistical analyses were performed using R version 4.3.0. The Wilcoxon signed-rank test was used for paired comparisons between baseline and 6 months. For longitudinal analyses, paired comparisons between baseline and each follow-up time point (3 and 6 months) were performed using the Wilcoxon signed-rank test, with *p*-values adjusted for multiple comparisons using the Bonferroni method. The Mann–Whitney U test and Fisher’s exact test were used for comparisons of continuous and categorical variables, respectively, between independent groups. All statistical tests were two-tailed, and a *p*-value < 0.05 was considered statistically significant.

## Results

### Clinical characteristics of MASLD with T2DM patients treated with 6-month imeglimin therapy

A total of 28 MASLD patients complicated with T2DM were included in this study (Table 1). Median age was 57.5 (IQR: 42.5–70.5) years, and 42.9% of patients were male. The prevalence of HT and HL was 46.4% and 89.3%, respectively. Median body weight was 74.2 (IQR: 66.2–87.4) kg, and median body mass index (BMI) was 28.83 (IQR: 24.30–32.98) kg/m^2^. Most patients were receiving background antihyperglycemic therapy, including SGLT2 inhibitors and GLP-1 receptor agonists, which remained stable throughout the study period.

**Table 1:** Comparison of clinical and laboratory data before and after 6-month imeglimin therapy

	Before	At 6 months	p-value
Age (years)	57.5 (42.5–70.5)		
Male	12 (42.9%)		
HT	13 (46.4%)		
HL	25 (89.3%)		
Metformin/DPP4/SGLT2-I/GLP1-RA/insulin	13 (46.4%)/5 (17.9%)/25 (89.3%)/14 (50%)/7 (25%)		
Body weight (kg)	**74.2 (66.2–87.4)**	**73 (63.5–86.8)**	**<0.001**
BMI (kg/m^2^)	**28.83 (24.30–32.98)**	**26.85 (23.99–33.20)**	**<0.001**
Body fat (%) #	**40.2 (37.7–44.6)**	**37.6 (30.3–39.5)**	**0.018**
Muscle mass (%) #	56.61 (52.23–58.76)	57.50 (55.38–59.79)	0.106
Skeletal muscle mass (%) #	31.93 (29.31–34.45)	32.58 (31.10–34.67)	0.083
ALB (g/dL)	4.5 (3.92–4.80)	4.45 (3.97–4.80)	0.586
UN (mg/dL)	14.5 (12.0–21.0)	16.0 (13.8–17.0)	0.614
Cre (mg/dL)	**0.70 (0.59–0.80)**	**0.71 (0.62–0.84)**	**0.031**
eGFR (mL/min/1.73 m^2^)	80.5 (67.4–90.0)	74.0 (65.3–86.3)	0.137
AST (U/L)	**33.0 (23.5–43.3)**	**26.0 (17.8–34.8)**	**<0.001**
ALT (U/L)	**28.5 (19.0–56.3)**	**23.5 (16.8–49.8)**	**0.001**
ALP (U/L)	80 (54–94)	69 (57–88)	0.067
GGT (U/L)	41.0 (25.5–63.0)	39.0 (22.5–58.0)	0.341
T-Bil (mg/dL)	0.60 (0.40–0.70)	0.60 (0.47–0.80)	0.091
PLT (× 10^4^/μL)	**21.50 (13.35–27.55)**	**23.80 (13.30–29.05)**	**0.042**
ALBI score	-3.16 (− 3.56–-2.79)	-3.20 (− 3.46–-2.62)	0.909
FBS (mg/dL)	**148.5 (126.0–193.5)**	**131.0 (114.0–149.8)**	**0.015**
HbA1c (%)	7.70 (6.77–8.20)	7.00 (6.50–7.53)	0.065
TC (mg/dL)	177 (159–210)	175 (140–206)	0.366
TG (mg/dL)	124.0 (101.0–195.0)	107.0 (81.5–210.0)	0.28
LDL-C (mg/dL)	**113.0 (86.0–125.5)**	**101.0 (66.5–114.0)**	**0.097**
HDL-C (mg/dL)	51.0 (39.3–55.0)	51.5 (43.0–54.8)	0.321

^#^Body composition parameters (body fat, muscle mass, and skeletal muscle mass) were available for a subset of patients (n = 15). Statistically significant values (*P* < 0.05) are highlighted in bold

ALB, Albumin; ALBI, Albumin-bilirubin; ALP, Alkaline phosphatase; ALT, Alanine aminotransferase; AST, Aspartate aminotransferase; BMI, Body mass index; Cre, Creatinine; DPP4, Dipeptidyl Peptitidase−4 inhibitor; eGFR, Estimated glomerular filtration rate; FBS, Fasting blood sugar; GGT, Gamma-glutamyltransferase; GLP1-RA, Glucagon-like peptide−1 receptor agonist; HbA1c, Hemoglobin A1c; HDL-C, High-density lipoprotein cholesterol; HL, Hyperlipidemia; HT, hypertension; LDL-C, low-density lipoprotein cholesterol; PLT, platelet count; SGLT2-I, sodium-glucose transporter 2 inhibitor; T-Bil, Total bilirubin; TC, Total cholesterol; TG, Triglyceride; UN, Urea nitrogen

### Changes in glycemic control and liver function after 6-month imeglimin therapy

**Fig. 1:**
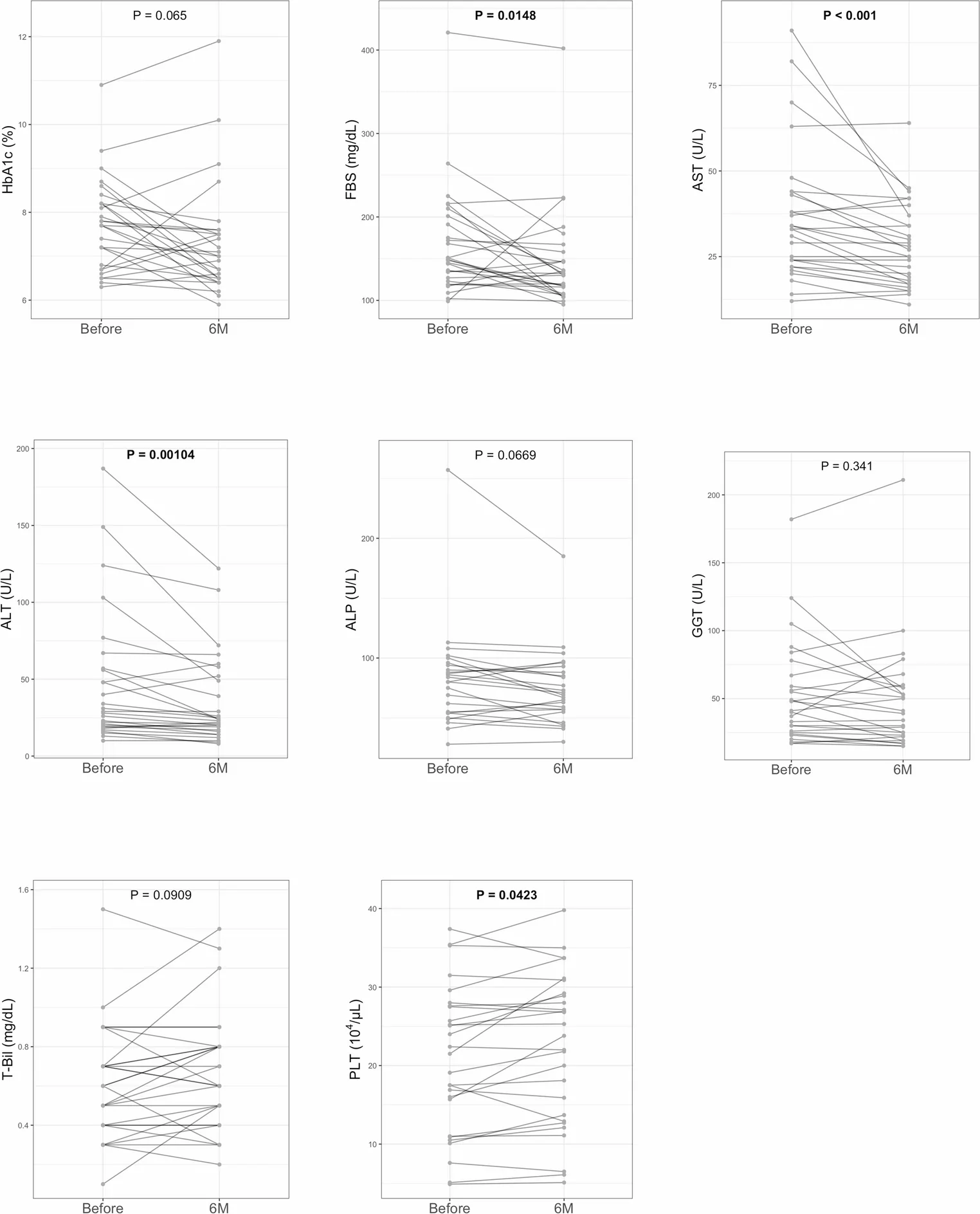
Changes in laboratory parameters at baseline and after 6 months (6M) of imeglimin therapy. ALBI, albumin-bilirubin; ALP, alkaline phosphatase; ALT, alanine aminotransferase; AST, aspartate aminotransferase; T-Bil, total bilirubin; FBS, fasting blood sugar; GGT, gamma-glutamyltransferase; HbA1c, hemoglobin A1c; PLT, platelet count

At 6 months, FBS levels were significantly reduced (148.5 [126.0–193.5] mg/dL to 131.0 [114.0–149.8] mg/dL, *p* = 0.015) (Table 1, Fig. 1). Serum aspartate aminotransferase (AST) (33 [23.5–43.3] U/L to 26 [17.8–34.8] U/L, *p* < 0.001) and ALT (28.5 [19.0–56.3] U/L to 23.5 [16.8–49.8] U/L, *p* = 0.001) levels also decreased. Platelet count (PLT) increased significantly from 21.50 (13.35–27.55) ×10^4^/µL to 23.80 (13.30–29.05) ×10^4^/µL (*p* = 0.042). No significant changes were noted for ALB, alkaline phosphatase (ALP), gamma-glutamyltransferase (GGT), T-Bil, blood urea nitrogen, or lipid parameters. Although not statistically significant, HbA1c showed a decreasing tendency (7.70 [6.77–8.20]% to 7.00 [6.50–7.53]%, *p* = 0.065). No major adverse events or treatment discontinuations were reported during the observation period, suggesting that imeglimin therapy was well tolerated and safe in this patient population.

### Changes in body composition parameters after 6-month imeglimin therapy

**Fig. 2:**
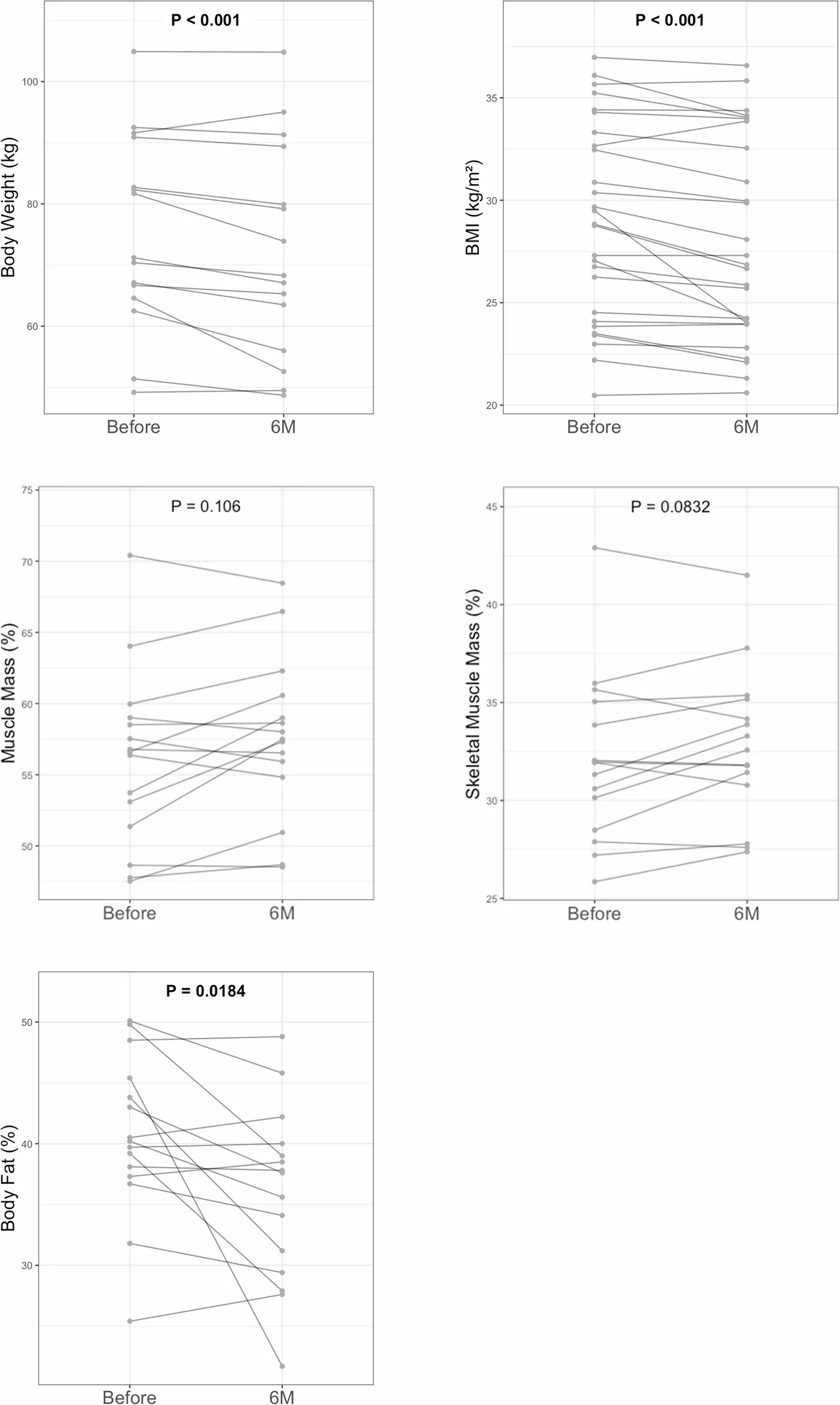
Changes in body weight, BMI, muscle mass, skeletal muscle mass, and body fat percentage in 15 patients at baseline and after 6 months (6M) of imeglimin therapy

To evaluate the effects of imeglimin on body composition, we analyzed the changes in related parameters at baseline and at 6 months of imeglimin therapy. As shown in Table 1 and Fig. 2, significant reductions in body weight (74.2 [66.2–87.4] kg to 73 [63.5–86.8] kg, *p* <0.001) and body fat (40.2 [37.7–44.6]% to 37.6 [30.3–39.5]%, *p* = 0.018) were observed. In contrast, no significant changes were noted for muscle mass (56.61 [52.23–58.76]% to 57.50 [55.38–59.79]%, *p* = 0.106) or skeletal muscle mass (31.93 [29.31–34.45]% to 32.58 [31.10–34.67]%, *p* = 0.083). These findings suggested that imeglimin therapy not only effectively lowered FBS levels, but also contributed to improvements in hepatic markers without remarkable muscle reduction. Baseline characteristics were compared between patients with and without body composition data. Most characteristics were broadly comparable, although FBS and ALP differed significantly between the groups (Supplementary Table 1).

### Longitudinal changes in glycemic and hepatic parameters during imeglimin therapy

**Fig. 3:**
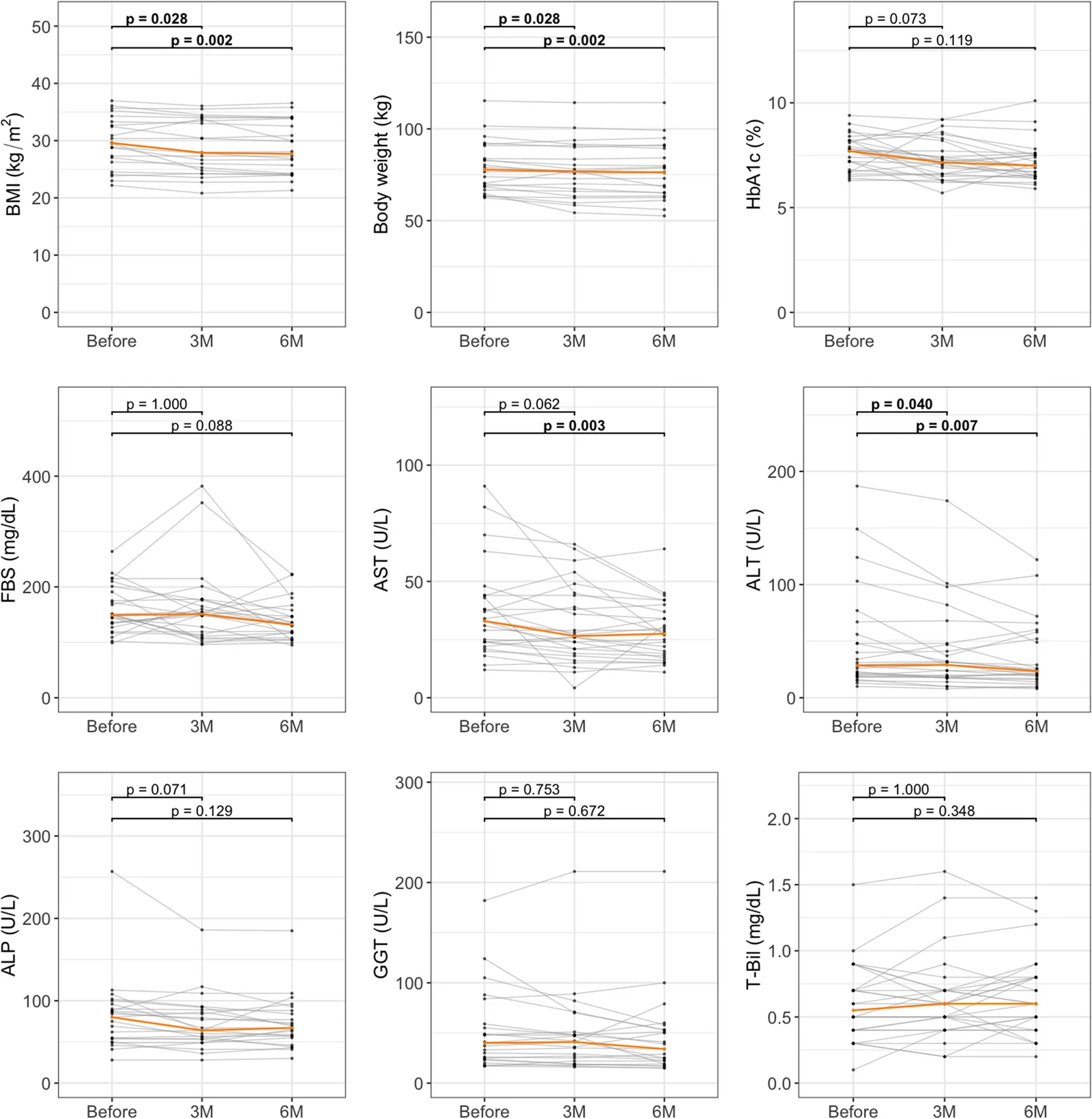
Longitudinal changes in metabolic and hepatic parameters during imeglimin therapy. Boxplots show HbA1c, FBS, body weight, BMI, AST, ALT, ALP, GGT, and T-Bil at baseline, 3 months (3M), and 6 months (6M)

Changes in key laboratory parameters were evaluated at baseline and at 3 and 6 months after initiation of imeglimin therapy (Fig. 3). FBS showed an overall numerical decrease during the 6-month treatment period, although the changes did not reach statistical significance after adjustment for multiple comparisons (3 months, *p* = 1.000; 6 months, *p* = 0.088). HbA1c also tended to decrease during follow-up (3 months, *p* = 0.073; 6 months, *p* = 0.119). ALT levels significantly decreased at both 3 and 6 months (*p* = 0.040 and *p* = 0.007, respectively), whereas AST levels showed a significant reduction at 6 months but not at 3 months (*p* = 0.062 and *p* = 0.003, respectively). Body weight and BMI significantly decreased at both 3 months (both *p* = 0.028) and 6 months (both p = 0.002). No significant changes in ALP, GGT, or T-Bil were observed at either time point.

### Subgroup analyses according to concomitant antidiabetic medications

Changes in liver enzymes did not differ significantly according to concomitant use of SGLT2 inhibitors (ΔALT, *p* = 0.823) or GLP-1 receptor agonists (ΔALT, *p* = 0.231). Background antidiabetic therapies remained stable throughout the study period. These analyses were exploratory and limited by the small sample size.

### Responder analysis based on Δ30% ALT improvement

To identify factors associated with improved liver enzyme levels, the cohort was stratified according to whether ALT levels improved remarkably by imeglimin therapy. Responders were defined as patients who showed a ≥30% reduction in ALT levels at 6 months (responders: n = 10; non-responders: n = 18). As shown in Table 2, baseline characteristics including age, BMI, HT, and HL did not differ significantly between the responders and non-responders. Responders tended to have poorer baseline glycemic control than non-responders (responder FBS: 196.0 [153.8–213.8] mg/dL vs. non-responder FBS: 140.0 [119.0–151.0] mg/dL, *p* = 0.023; responder HbA1c: 8.25 [7.82–8.67]% vs. non-responder HbA1c: 7.20 [6.70–7.70]%, *p* = 0.012). No other biochemical parameters exhibited significant differences between the responder and non-responder groups, although responders tended to have lower body fat (32.90 [28.03–38.28]% vs. 40.35 [38.38–45.27]%, *p* = 0.079) and higher TG (196.0 [132.8–293.3] mg/dL vs. 121.0 [97.3–145.5] mg/dL, *p* = 0.089) at baseline.

**Table 2:** Comparison of baseline clinical and laboratory characteristics between responders and non-responders after 6-month imeglimin therapy

	Responders(n = 10)	Non-responders(n = 18)	p-value
Age (years)	55.50 (41.50–57.75)	62.00 (52.00–71.50)	0.457
Male	4 (40.0%) / 6 (60.0%)	12 (66.7%) / 6 (33.3%)	0.243
HT	6 (60.0%) / 4 (40.0%)	9 (50.0%) / 9 (50.0%)	0.705
HL	1 (10.0%) / 9 (90.0%)	2 (11.1%) / 16 (88.9%)	1.000
Body weight (kg)	72.05 (64.89–82.92)	75.25 (65.95–88.98)	1.000
BMI (kg/m^2^)	26.50 (23.52–32.40)	29.13 (26.38–32.60)	0.382
Body fat (%)	32.90 (28.03–38.28) #	40.35 (38.38–45.27) $	0.079
Muscle mass (%)	63.45 (58.41–67.98) #	56.49 (51.46–58.27) $	0.079
Skeletal muscle mass (%)	36.25 (33.04–39.47) #	31.63 (28.60–34.29) $	0.158
ALB (g/dL)	4.5 (4.5–4.7)	4.2 (3.9–4.8)	0.402
UN (mg/dL)	12 (11–17)	15.5 (12–21)	0.333
Cre (mg/dL)	0.73 (0.64–0.79)	0.70 (0.58–0.78)	0.782
eGFR (mL/min/1.73 m^2^)	83.2 (75.7–101.5)	71.1 (62.9–82.6)	0.120
AST (U/L)	32 (21.8–61.0)	33.5 (24.0–41.8)	0.981
ALT (U/L)	52.0 (21.5–91.5)	27.0 (19.0–38.5)	0.231
ALP (U/L)	69 (55–80)	86 (54–94)	0.434
GGT (U/L)	26.0 (23.0–88.0)	44.5 (30.8–55.8)	0.837
T-Bil (mg/dL)	0.55 (0.43–0.70)	0.60 (0.40–0.70)	1.000
PLT (× 10^4^/μL)	18.3 (16.4–27.9)	22.4 (10.9–25.7)	0.744
ALBI score	− 3.16 (− 3.32–-3.00)	− 2.85 (− 3.61–-2.56)	0.706
FBS (mg/dL)	**196.0 (153.8–213.8)**	**140.0 (119.0–151.0)**	**0.023**
HbA1c (%)	**8.25 (7.82–8.67)**	**7.20 (6.70–7.70)**	**0.012**
TC (mg/dL)	172 (148–226)	194 (168–209)	0.952
TG (mg/dL)	196.0 (132.8–293.3)	121.0 (97.3–145.5)	0.089
LDL-C (mg/dL)	93.5 (70.7–130.3)	114.5 (98.5–126.3)	0.472
HDL-C (mg/dL)	52.0 (47.8–58.0)	50.0 (39.0–55.0)	0.406

Responders: patients who achieved a ≥ 30% reduction in ALT levels at 6 months

Non-responders: patients who did not achieve a ≥ 30% reduction in ALT levels at 6 months

Body composition parameters (body fat, muscle mass, and skeletal muscle mass) were available in 4 responders (#) and 11 non-responders ($). Statistically significant values (*P* < 0.05) are highlighted in bold

ALB, Albumin; ALBI, Albumin-bilirubin; ALP, Alkaline phosphatase; ALT, Alanine aminotransferase; AST, Aspartate aminotransferase; BMI, Body mass index; Cre, Creatinine; eGFR, Estimated glomerular filtration rate; FBS, Fasting blood sugar; GGT, Gamma-glutamyltransferase; HbA1c, Hemoglobin A1c; HDL-C, High-density lipoprotein cholesterol; HL, Hyperlipidemia; HT, Hypertension; LDL-C, Low-density lipoprotein cholesterol; PLT, Platelet count; T-Bil, Total bilirubin; TC, Total cholesterol; TG, Triglyceride; UN, Urea nitrogen

## Discussion

### Main findings

This study demonstrated early and sustained changes in metabolic and hepatic parameters in patients with MASLD and T2DM treated with imeglimin. Within 6 months, fasting glucose, transaminase levels, and body fat percentage decreased, accompanied by an increase in platelet count. In the longitudinal analysis, significant reductions in ALT, body weight, and BMI were observed from 3 months, whereas AST significantly decreased at 6 months. Importantly, no statistically significant changes were observed in lean mass, including skeletal muscle mass. Taken together, these findings suggest a potential metabolic and hepatic effect of imeglimin and may be relevant for patients with MASLD, particularly those at risk of sarcopenia.

### Context with published literature

MASLD is closely associated with insulin resistance and T2DM [30]. While anti-diabetic agents such as SGLT2 inhibitors and GLP-1 receptor agonists have shown beneficial effects on hepatic steatosis [13, 31, 32], preservation of skeletal muscle mass has emerged as an important clinical issue in MASLD, particularly in elderly patients and those at risk of sarcopenia [33–37]. Characterized by hepatic steatosis in individuals with BMI within the non-obese range, so-called “lean MASLD” is increasingly being recognized, particularly in Asian populations [38–40]. Sarcopenia is also a frequent and clinically relevant complication in MASLD, sharing the common pathophysiological mechanisms of insulin resistance and physical inactivity [41, 42]. As sarcopenia is associated with more advanced fibrosis and worse clinical outcomes [20], therapeutic strategies that can improve liver function while preserving skeletal muscle mass are highly desirable.

Imeglimin is a novel oral agent that ameliorates glycemic control through mitochondrial mechanisms affecting both pancreatic β-cell function and insulin sensitivity in peripheral tissues [43]. Recent preclinical and clinical studies suggest that imeglimin may also exert hepatoprotective effects, including the reduction of inflammation and fibrosis [19, 44, 45]. In mice fed a high-fat, high-sucrose diet, imeglimin improved fatty liver findings and enhanced glucagon secretion through an indirect mechanism [45]. Imeglimin has been reported to modulate hepatic lipid metabolism through two mechanisms: a pancreatic effect that promotes glucose concentration-dependent insulin secretion in pancreatic β cells through its effects on the NAMPT (NAD+ synthesis enzyme) gene and mitochondrial respiratory chain complex I, as well as an extrapancreatic effect that ameliorates glucose metabolism in the liver and skeletal muscle by gluconeogenesis inhibition and enhanced glucose uptake, potentially contributing to reduced steatosis [19, 43, 44]. The poorer baseline glycemic control observed in ALT responders may reflect greater glucotoxicity and insulin resistance and, consequently, greater potential for metabolic improvement with imeglimin. Through these metabolic and mitochondrial actions [17, 18], improved glycemic control may in turn contribute to reduced hepatic injury.

Consistent with our findings, a recent multicenter study by Fukunaga et al. reported that imeglimin significantly improved transaminase levels in patients with T2DM and MASLD, supporting a potential hepatoprotective effect of this agent [46]. Importantly, normal or near-normal ALT levels do not exclude clinically significant liver disease. In biopsy-proven NAFLD, patients with low-normal ALT levels have been shown to have less severe steatosis and perisinusoidal fibrosis than those with high-normal ALT levels [47], while clinically significant NASH and advanced fibrosis may still be present despite normal aminotransferase levels [48, 49]. Thus, the consistent reduction in ALT observed at both 3 and 6 months, despite a median baseline ALT of only 28.5 U/L, may represent a modest but reproducible biochemical improvement. This interpretation is consistent with current AASLD guidance, which recognizes improvement in ALT in response to an intervention as a potential surrogate marker of histological improvement in disease activity [50].

In the present study, imeglimin therapy was likewise accompanied by favorable changes in glycemic and hepatic parameters, together with a modest but significant reduction in body weight and fat mass. The approximately 1.2-kg weight loss was consistent with previous clinical studies of imeglimin [51] and occurred without a statistically significant reduction in skeletal muscle mass. Although this magnitude of weight reduction is unlikely, in isolation, to indicate histological improvement or fibrosis regression, it may still be clinically relevant in patients with MASLD, in whom preservation of muscle mass is an important consideration. Such modest reductions in body weight and visceral adiposity may contribute, at least in part, to changes in hepatocellular injury markers and inflammatory activity mediated by enhanced insulin sensitivity and reduced lipotoxic stress, as reflected by changes in serum transaminase levels [52], even if they do not translate into demonstrable structural remodeling.

To date, no head-to-head trials have directly compared imeglimin with GLP-1 receptor agonists or SGLT2 inhibitors with respect to liver enzymes and body composition; therefore, their relative effects can only be discussed indirectly. While GLP-1 receptor agonists and SGLT2 inhibitors generally induce greater weight loss, such effects may be accompanied by a potential reduction in lean mass [53]. In the present study, imeglimin was associated with modest improvements in transaminase levels together with a small reduction in fat mass and no statistically significant reduction in skeletal muscle mass, a profile that may be clinically relevant for patients with MASLD, particularly those at risk of sarcopenia.

### Strengths and limitations

A major strength of this study is the comprehensive evaluation of both hepatic parameters and body composition in patients with MASLD and type 2 diabetes treated with imeglimin. The inclusion of serial assessments at 3 and 6 months enabled characterization of time-dependent changes in liver enzymes as well as fat and muscle mass, which is particularly relevant in patients with MASLD at risk of sarcopenia. The use of body composition analysis allowed us to evaluate changes in glycemic and hepatic parameters without a statistically significant reduction in skeletal muscle mass.

Several limitations should be acknowledged. First, adherence to medication and lifestyle recommendations was not objectively assessed, and their potential influence on the observed outcomes cannot be excluded. Second, the retrospective single-arm design and small sample size limit causal inference and statistical power; exploratory analyses, including the responder analysis, should therefore be regarded as hypothesis-generating. Third, treatment efficacy was assessed using serum ALT rather than histological endpoints. Although changes in ALT may reflect disease activity, they do not provide direct evidence of histological improvement or long-term clinical benefit, and the optimal ALT response threshold in patients with near-normal baseline ALT levels remains uncertain. Fourth, body composition was assessed using bioelectrical impedance analysis, which is less precise than reference-standard methods such as DXA or MRI and may be influenced by hydration status and measurement conditions [54].

### Future implications

To validate and extend the present findings, future studies should adopt prospective, large-scale designs with longer follow-up. Incorporation of histological assessment through liver biopsy or validated non-invasive biomarkers, together with reference-standard modalities such as dual-energy X-ray absorptiometry or magnetic resonance imaging for body composition, will provide more definitive insights into the hepatic and musculoskeletal effects of imeglimin. In particular, inclusion of patients with higher baseline liver enzyme levels or active hepatic inflammation may enable a clearer evaluation of treatment responsiveness.

Given the observed changes in glycemic parameters and liver enzyme levels without a statistically significant reduction in skeletal muscle mass, imeglimin may represent a potential therapeutic option for vulnerable patient populations, particularly those with lean MASLD and elderly individuals at risk of sarcopenia. These findings suggest that imeglimin may be associated with metabolic and hepatic effects while maintaining lean body mass, although further validation in prospective studies is required.

## Supplementary Information

Below is the link to the electronic supplementary material.

Supplementary file1 (DOCX 22 KB)

## Data Availability

Study data will be provided by the corresponding author upon reasonable request.

## Abbreviations

ALB: Albumin
ALBI: Albumin-bilirubin
ALP: Alkaline phosphatase
ALT: Alanine aminotransferase
BMI: Body mass index
eGFR: Estimated glomerular filtration rate
FBS: Fasting blood sugar
GGT: Gamma-glutamyltransferase
GLP-1: Glucagon-like peptide-1
HbA1c: Hemoglobin A1c,
HL: Hyperlipidemia
HT: Hypertension
IQR: Interquartile range
MASLD: Metabolic dysfunction-associated steatotic liver disease
NAFLD: Non-alcoholic fatty liver disease
PLT: Platelet count
SGLT2: Sodium-glucose cotransporter 2
TG: Triglyceride
T2DM: Type 2 diabetes mellitus
T-Bil: Total bilirubin
