# Supplementary material for: Effects of imeglimin on liver enzymes and body composition in patients with MASLD and type 2 diabetes: a pilot study
Source: Diabetol Int. 2026 Oct 5;17(4):80. doi: 10.1007/s13340-026-00931-4 (PMC13635075; doi:10.1007/s13340-026-00931-4)
Supplement: Supplementary file 1 [file 13340_2026_931_MOESM1_ESM.docx]

**Supplementary Table 1. Comparison of Baseline Characteristics Between Patients With and Without Body Composition Assessment**

|  | Patients with BIA  (n = 15) | Patients without BIA  (n = 13) | p-value |
| --- | --- | --- | --- |
| Age (years) | 60.00 (55.30–71.50) | 54.00 (40.00–68.00) | 0.407 |
| Male | 4 (26.6%) / 11 (73.4%) | 8 (61.5%) / 5 (38.5%) | 0.125 |
| HT | 7 (46.7%) / 8 (53.3%) | 6 (46.2%) / 7 (53.8%) | 1.000 |
| HL | 14 (93.3%) / 1 (6.7%) | 11 (84.6%) / 2 (15.4%) | 0.583 |
| Body weight (kg) | 70.40 (64.88–88.98) | 74.20 (67.20–86.83) | 0.661 |
| BMI (kg/m²) | 29.67 (26.38–32.60) | 27.30 (23.92–33.56) | 0.475 |
| ALB (g/dL) | 4.60 (3.93–4.80) | 4.50 (3.95–4.85) | 0.938 |
| AST (U/L) | 33.00 (24.30–43.80) | 31.00 (20.30–46.00) | 0.475 |
| ALT (U/L) | 29.00 (19.50–54.00) | 26.00 (18.30–68.50) | 0.981 |
| **ALP (U/L)** | **90.00 (60.50–101.50)** | **62.00 (49.30–78.80)** | **0.023** |
| GGT (U/L) | 41.00 (25.30–58.00) | 40.50 (26.50–83.00) | 0.845 |
| T-Bil (mg/dL) | 0.60 (0.33–0.90) | 0.60 (0.48–0.70) | 0.889 |
| PLT (×10⁴/μL) | 24.00 (10.93–27.05) | 17.50 (15.85–27.80) | 0.732 |
| **FBS (mg/dL)** | **127.00 (117.50–149.50)** | **201.00 (148.80–218.30)** | **<0.001** |
| HbA1c (%) | 7.20 (6.63–8.10) | 7.90 (7.58–8.78) | 0.055 |

ALB, albumin; ALP, alkaline phosphatase; ALT, alanine aminotransferase; AST, aspartate aminotransferase; BMI, body mass index; FBS, fasting blood sugar; GGT, gamma-glutamyltransferase; HbA1c, hemoglobin A1c; HL, hyperlipidemia; HT, hypertension; PLT, platelet count; T-Bil, total bilirubin.
